# Transformation of an Unclassified Myeloproliferative Neoplasm with a Rare *BCR-JAK2* Fusion Transcript Resulting from the Translocation (9;22)(p24;q11)

**DOI:** 10.1155/2015/252537

**Published:** 2015-02-18

**Authors:** A. N. Chamseddine, P. Etancelin, D. Penther, F. Parmentier, C. Kuadjovi, V. Camus, N. Contentin, P. Lenain, C. Bastard, H. Tilly, F. Jardin

**Affiliations:** ^1^Department of Clinical Hematology, Henri Becquerel Cancer Center, 1 rue d'Amiens, 76038 Rouen, France; ^2^Blood and Marrow Transplant Unit, Henri Becquerel Cancer Center, 1 rue d'Amiens, 76038 Rouen, France; ^3^Molecular and Genetic Laboratory Department, Henri Becquerel Cancer Center, 1 rue d'Amiens, 76038 Rouen, France; ^4^INSERM U918 Unit, Henri Becquerel Cancer Center, 1 rue d'Amiens, 76038 Rouen, France

## Abstract

*BCR-ABL1* negative myeloproliferative neoplasms (MPNs) are known to contain alterations of the tyrosine kinase JAK2 (located on 9p24) that result in constitutive activation of the encoded protein. JAK2 fusions are reported in acute and chronic leukemias of myeloid and lymphoid phenotypes. Here, we report an unclassified case of MPN (MPN-U) showing a t(9;22)(p24;q11), which generates a *BCR-JAK2* fusion gene by fusing the *BCR* at intron 13 to *JAK2* at intron 17 on the derivative chromosome 22. Most reported JAK2 fusions cases reveal an aggressive clinical course and long-term remissions have only been achieved after allogeneic stem cell transplantation (ASCT). To the best of our knowledge, this is the thirteenth case reported worldwide to describe a *BCR-JAK2* fusion transcript in MPN-U. The present report revealed a sustained complete clinical, hematologic, and cytogenetic remission 35 months after diagnosis and ~24 months after ASCT. Regarding *BCR-ABL1* *negative* MPN patients this case report provides strong support for a role of *JAK2* activation in the oncogenesis and suggests a possible diagnostic and therapeutic target that should be investigated.

## 1. Introduction

Some myeloproliferative neoplasms (MPNs) are Philadelphia- (Ph-) negative, lacking the reciprocal t(9;22)(q34;q11) and its resultant* BCR-ABL1* fusion gene. Currently, the most frequent genomic abnormality observed in Ph-negative MPN is a dominant gain-of-function V617F mutation in the JH2 kinase-like domain of* JAK2* [1]. However, there are rare additional mechanisms described in Ph-negative MPNs that activate* JAK2*, such as chromosomal translocations that cause constitutive dimerization through the replacement of amino terminal sequences with a fusion partner [2, 3]. Indeed six different fusion partners have been associated with* JAK2* (*RPN1, SSBP2, PAX5, PCM1, BCR,* and* ETV6*).

Here, we report a rare case of unclassified MPN (MPN-U) with a t(9;22)(p24;q11) leading to a* 5*′*BCR*/*3*′*JAK2* fusion gene producing a fusion transcript that juxtaposed* BCR* exon 13 and* JAK2* exon 17 and subsequently rapidly transformed into a myeloid granulocytic sarcoma. We also describe, 35 months after diagnosis and ~24 months after ASCT, a prolonged and sustained complete clinical, hematologic, and cytogenetic remission after undergoing allogeneic stem cell transplantation (ASCT).

## 2. Case Presentation

**Figure 1:**
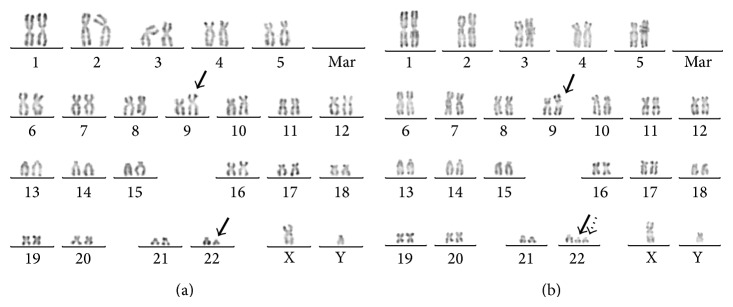
Conventional cytogenetics (bone marrow) analysis revealing: (a) 46, XY, t(9;22)(p24;q11) in 60% of the metaphases: arrows indicate the balanced translocation between the short arm of the chr9 and the long arm of the chr22. (b) 47, XY, t(9;22)(p24;q11), +der(22) t(9;22)(q24;q11) in 10% of the metaphases: arrows indicate the chr9 and chr22 involved in the t(9;22)(p24;q11) and dotted arrow indicates the duplication of derived.

We report a case of a 49-year-old man with no significant medical history. The patient was referred to our center in October 2011. The blood count was abnormal with anemia (Hb 11.2 g/dL) and a platelet count of 78000/mm^3^. The white blood cell count was 11500/mm^3^ with 30% lymphocytes, 2% monocytes, 2% eosinophils, 0% basophils, 29% neutrophils, and 37% promyelocytes, myelocytes, and metamyelocytes. Clinical examination was unremarkable. The bone marrow aspiration and biopsy associated with initial molecular blood and medullary analyses led to diagnose an MPN-U. It did not reveal any BCR-ABL1 rearrangement neither V617F JAK2 mutation. In February 2012, the patient presented to the emergency room with a sudden onset of pyramidal tract deficiency syndrome and with an increase of leukocytosis and blood myeloid precursors. The MRI scan revealed a thoracic spinal epidural compression extending from T4 to T10. Emergent laminectomy was done. Histological analysis was performed on the laminectomy specimen and demonstrated the presence of a granulocytic (myeloid) sarcoma. Radiation therapy was then performed. Cytogenetic examination of the bone marrow aspiration of the patient was performed on two unstimulated short-term cultures (24 hrs and 48 hrs). The karyotype was obtained by conventional R-banding analysis [4]. Chromosome analysis (Figure 1) showed t(9;22)(p24;q11) as the sole abnormality in 60% of the analyzed metaphases (12/20). In 10% of the analyzed metaphases (2/20), it showed the latter translocation in addition to der(22) t(9;22)(p24;q11). The last 30% of the analyzed metaphases (6/20) were normal. Mutations of exons 12, 13, and 14 and, in particular, the V617F JAK2 gene mutation were not found. Considering the t(9;22)(p24;q11), that the exons 12, 13, and 14 and the V617F* JAK2* mutations were absent, and that* JAK2* had previously been shown to fuse with* BCR* in MPN-like patients, the best fusion gene candidates were* JAK2* in 9p24 and* BCR* in 22q11.

**Figure 2:**
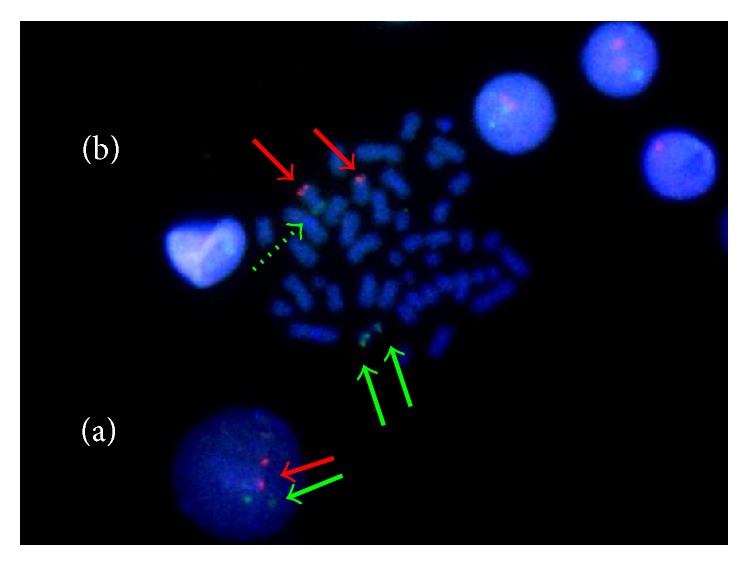
FISH analysis. (a) Nucleus with two red signals (red arrow, ABL probe) on chr9 and two green signals (green arrow, BCR probe) on chr22, indicating that BCR-ABL1 FISH for the Ph-chromosome did not reveal any fusion signals, revealing a normal hybridization pattern negative for t(9;22)(q34;q11.2) BCR-ABL1 fusion. (b) Two red signals (red arrows, ABL probe) were present on the long arm of both the normal and the derivative chr9. Three green signals were present, indicating an extra signal of the BCR probe, suggestive of an extra chromosome 22 or additional chromosome material containing the 22q11.2 region: two intense green signals were on the normal chr22 (green arrows, BCR probe) and one reduced intensity green signal was localized on the derivative chr22 and on the short arm of derivative chr9 (dotted green arrow, telomeric part of the BCR probe). Notice that one of the derivative chromosome signals was too feint to be seen in some nuclei.

FISH analysis (Figure 2) using dual fusion probes for* BCR* (22q11.2) and* ABL1* (9q34) regions (LSI* BCR*/*ABL* ES Dual Color Translocation Probe, Abbott Molecular; Vysis, Des Plaines, IL, USA) excluded the* BCR-ABL1* fusion and showed an extra signal of the* BCR* probe on chromosome 9p. Moreover, a FISH assay was performed using* JAK2* probes obtained from bacterial artificial chromosome (BAC) (RP11-39K24 AL161450, Sanger Institute, Cambridge, UK) labelled with spectrum red (Vysis, Downers Grove, IL, USA) and showed a split of the probe between 9p24 and 22q11. According to the cytogenetic examination and FISH results which showed a t(9;22)(p24;q11), a* BCR-JAK2* fusion was suggested.

**Figure 3:**
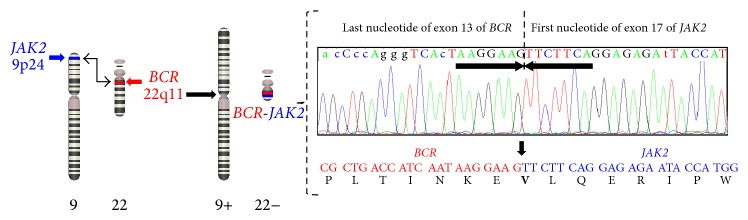
Mechanism of* BCR-JAK2* fusion and breakpoints direct sequencing. Sanger sequencing alignment of the RT-PCR product revealed a break at nucleotide 3458 of exon 13 of* BCR* (nucleotides highlighted in red) and at nucleotide 1 of exon 17 of* JAK2* (nucleotides highlighted in blue). Amino acids of the respective fusion gene* BCR-JAK2* reveal a new valine residue (V) that has been created at the fusion junction.

Blood from the patient was collected in EDTA, and RNA was isolated from 107 cells using the TRIzol kit (Life Technologies, Carlsbad, CA, USA). cDNA synthesis was performed using Moloney Murine Leukemia Virus reverse transcriptase (Invitrogen, Life Technologies, Carlsbad, CA, USA). The breakpoint Sanger sequencing using ABI 3130 (Life Technologies, Carlsbad, CA, USA) identified an in-frame fusion of the last nucleotide of BCR exon 13 with the first nucleotide of JAK2 exon 17 (Figure 3). There was no loss or insertion of a base at these breakpoints.

**Figure 4:**
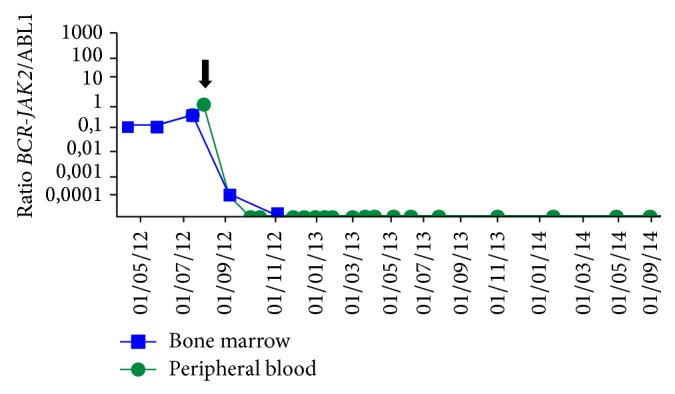
Quantitative real time PCR expression levels of* BCR-JAK2* follow-up.* BCR-JAK2* follow-up carried out relative to the expression of the housekeeping gene ABL1, in the bone marrow and the peripheral blood, after allogeneic stem-cell transplantation (black arrow). It revealed one of the longest sustained complete hematologic and cytogenetic remissions at 35 months of follow-up in a* BCR-JAK2* fusion MPN-U.

To follow up the minimal residual disease, a specific primer-probe assay was designed. Real time quantitative PCR (QPCR) using TaqMan chemistry was performed on an Applied 7500 (Life Technologies, Carlsbad, CA, USA) with the following sequences: forward primer 5′-GCT GAC CAA CTC GTG TGT GAA-3′, reverse primer 5′-TCA GGT GGT ACC CAT GGT ATT CT-3′ and the probe FAM 5′-CAG CAT TCC GCT GAC CAT CAA TAA GGA-3′. QPCR expression levels of* BCR-JAK2* were carried out relative to the expression of the housekeeping gene* ABL1*. Molecular monitoring was able to detect low levels of disease. Hence, the assay was >4 logs more sensitive than conventional cytogenetic, detecting one copy of* BCR-JAK2* to 10000 copies of* ABL1* (0.0001%) and allowing us to follow up the effectiveness of treatment. The patient underwent acute myeloid leukemia-like chemotherapy induction and consolidation achieving a chronic phase in May 2012. An ASCT from a matched human leukocyte antigen- (HLA-) unrelated donor (MUD) was then undertaken in August 2012 with a TBI-Endoxan regimen conditioning and without any Graft-versus-host disease complications. The QPCR follow-up of* BCR-JAK2* expression in both the bone marrow and peripheral blood mononuclear cells showed complete hematological and molecular (<0.0001%) remission 3 months later. With 35-month follow-up, the patient remains alive with undetectable* BCR-JAK2* transcript levels in the blood and no transplant-related complications (Figure 4).

## 3. Discussion

We have described the presence of a* BCR-JAK2* fusion gene in a patient with a rapid blast evolution. This fusion gene is the result of a reciprocal translocation between chr9 and chr22, implying the possible occurrence of a double break on chr9. A fragment of the 3′ end of exon 17 of the* JAK2* on chr9 translocated to exon 13 of chr22 in the proper orientation to generate an in-frame fusion transcript with the 5′ end of the* BCR* gene. The resultant encoded 1330-amino-acid chimeric protein contained the N-terminal coiled-coil dimerization domain of BCR and the C-terminal tyrosine kinase domain JH2 of JAK2. The constitutive activation of this chimeric protein is mediated by oligomerization through the coiled-coil domain of BCR and by disruption of the autoinhibitory role of the inhibitory regions (IR) of the pseudo-kinase domain JH2 of JAK2. In fact, there are three inhibitory regions (IR1, -2, and -3) within JH2. IR3, at the C terminus of JH2, directly inhibits JH1. IR2, in the C-terminal lobe of JH2, and IR1, extending from the N-terminal to the C-terminal lobe, enhance the IR3-mediated inhibition of JH1. Hence, the disruption of IR by mutation, deletion, or translocation increases basal JAK2 activity. Consequently, the BCR-JAK2 chimeric protein is entirely or partially deprived of IR1, which may result in the upregulation of JAK2 activity [5]. Preclinical studies implied a possible role of c-ABL1 in Jak2 activation in various Ph-negative myeloid malignancies [6] and demonstrated that the* BCR-JAK2* fusion gene induces* STAT5* activation and inhibits* BCRxL* gene expression, thereby promoting tumorigenic properties and increasing cell survival [7].

It was difficult to define the best therapy. JAK2 inhibitors alone or in combination with chemotherapy may not be effective against the* BCR-JAK2* fusion gene malignancies [7, 8]. Compared with* JAK2* mutations,* JAK2* fusions are probably associated with more aggressive diseases such as acute leukemias (myeloid or lymphoblastic), atypical CML (aCML), and myelofibrosis [9]. In our review of literature (Table 1) seven patients presented with aCML/MPN-U, one patient presented with AML, and one patient with ALL. As it is usually observed in myeloid neoplasms, a predominance of the male gender is reported. Three of the reported patients were unsuccessfully treated with tyrosine kinase inhibitors (imatinib or ruxolitinib). In addition, it is important to note that the mortality rate was 50% in cases where the follow-up was described. In our opinion, ASCT is likely the only curative strategy in these MPN-Us. In fact,* JAK2* translocations have been described in acute and chronic leukemias of myeloid and lymphoid phenotypes. Hence, we can hypothesize that MPN-U associated with rearrangements of* JAK2* is a hematopoietic stem cell (HSC) disease that is only curable by HSC transplantation [10].

## 4. Conclusion

We described a rare Ph-negative case of MPN with a* BCR-JAK2* transcript and a reciprocal t(9;22)(q34;q11) that was detected juxtaposing* BCR* exon 13 and* JAK2* exon 17. This rare entity underlies often an aggressive clinical course with rapid progression to blast phase within the first 2 years after diagnosis (Table 1). To the best of our knowledge, this is the thirteenth case reported worldwide. Furthermore we report here the first described isoform fusion transcript juxtaposing* BCR* exon 13 and* JAK2* exon 17. It revealed one of the longest sustained complete clinical, hematologic and cytogenetic remissions in a* BCR-JAK2* fusion MPN-U. These rare* BCR*-*JAK2* fusions suggest common pathways between* JAK2* activation and the natural history of lympho/myeloproliferative hematologic malignancies. One should take into consideration* JAK2* fusions when investigating Ph-negative MPN patients.

## Figures and Tables

**Table 1:** Characteristics of cases reported in the literature with *BCR-JAK2* fusion gene.

Reference	Year	Age	Sex	Translocation	Isoform	Clinical presentation	Treatment	Follow-up (FU)
Griesinger et al. [11]	2005	63	F	t(9;22) (p24;q11.2)	BCR exon 1 fused to JAK2 exon 19	aCML	Hy; Cy; Mit	Death from blast crisis
Cirmena et al. [12]	2008	67	F	t(9;22) (p24;q11)	BCR exon 14 fused to JAK2 exon 11	AML	HD + ASCT(MSD)	Death from disease relapse
Lane et al. [13]	2008	44	M	t(9;22) (p24;q11.2)	BCR exon 1 fused to JAK2 exon 17	aCML	ND	ND
Elnaggar et al. [14]	2012	84	M	t(9;22) (p24;q11.2)	BCR exon 1 fused to JAK2 exon 19	aCML	Hy; IM	ND
Tirado et al. [15]	2010	14	M	t(9;22) (p24;q11.2)	ND	ALL	Polychemotherapy, ASCT(MSD)	CHR at 6-month FU
Bellesso et al. [16]	2013	54	M	t(9;22) (p24;q11.2)	ND	aCML	IM, DAS + Hy, ASCT(MSD)	Death from aGVHD
Xu et al. [17]	2013	28	M	ins(22;9) (q11;p13p24)	BCR exon 1 fused to JAK2 exon 19	aCML	Hy + INF *α*	CHR at 27-month FU
Impera et al. [18]	2011	84	M	t(9;18;22) (p23;p11.3;q11.2)	BCR exon 1 fused to JAK2 exon 15	MPN-U	IM, DAS, INF *α*	CHR at 21-month FU
Schwaab et al. [8]	2015	ND	M	t(9;18) (p24;q12)^*^	BCR exon 1 fused to JAK2 exon 17	aCML	Jak2 inh	Relapse at 18-month FU
Cuesta-Domínguez et al. [7]	2012	58	M	49, XY, +X, +2, +4, 9, 11, +19, add(19) (q13), +20,22, +mar	BCR exon 1 fused to JAK2 exon 15	ALL	High-risk ALL protocol, ASCT, INF *α*	>6 years
Roberts et al. [19]	2012	2.7	M	+2, del(2) (p23), t(3;22;9) (p12;q11.2;p24)	BCR exon 1 fused to JAK2 exon 15	ALL	ND	ND
Angelova et al. [20]	2011	53	M	t(9;22) (p24;q11.2) as part of complex karyotype	ND	MPN-U	No treatment	Death from blast crisis
Present case	2011	49	M	t(9;22) (p24;q11)	BCR exon 13 fused to JAK2 exon 17	MPN-U/GS	3 + 7, ASCT(MUD)	CMR at 35-month FU

F: female; M: male; aCML: atypical chronic myeloid leukemia; AML: acute myeloid leukemia; ALL: acute lymphoblastic leukemia; MPN-U: unclassified myeloproliferative neoplasm; GS: granulocytic sarcoma; Hy: hydroxyurea; Cy: cytarabine; Mit: mitoxantrone; HD: high-dose chemotherapy; ASCT: allogeneic stem cell transplantation; ND: not described; MSD: matched sibling donor; MUD: matched unrelated donor; IM: imatinib; DAS: dasatinib, Jak2 inh: JAK1/JAK2 inhibitor ruxolitinib; aGVHD: acute graft-versus-host disease; CHR: complete hematological response; CMR: complete molecular remission. ^*^The RNA sequencing indicated the presence of a *BCR-JAK2* fusion gene. The BCR-JAK2 fusion was subsequently confirmed by RT-PCR and PCR from genomic DNA. *BCR-JAK2* in this case is therefore likely to be the result of a small insertion of BCR into the JAK2 locus on the der(18).
